# A BOPPPS-based micro-lecture teaching intervention for orthopaedic postgraduates in China

**DOI:** 10.1080/10872981.2026.2616194

**Published:** 2026-01-13

**Authors:** Xu Yang, Tianyang Liu, Ying Li, Fuqiang Gao

**Affiliations:** a Peking University China-Japan Friendship School of Clinical Medicine, Beijing, People's Republic of China; b Capital Medical University China-Japan Friendship Hospital School of Clinical Medicine, Beijing, People's Republic of China; c Educational Administration Department, China-Japan Friendship Hospital, Chaoyang District, Beijing, People's Republic of China; d Centre for Osteonecrosis and Joint-preserving & Reconstruction, Department of Orthopaedics, China-Japan Friendship Hospital, Chaoyang District, Beijing, People's Republic of China

**Keywords:** Micro-lectures, BOPPPS, practical teaching, orthopaedics research, postgraduate education

## Abstract

**Background:**

Orthopaedics is a highly specialized discipline characterized by complex theoretical frameworks and extensive knowledge, posing challenges for postgraduate teaching. With the advancement of educational technology, micro-lectures have been increasingly adopted in medical education. However, their isolated use may be insufficient to sustain student engagement or ensure effective learning. The bridge-in, learning objective, pre-test, participatory learning, post-assessment, and summary (BOPPPS) teaching model, which emphasizes the closed-loop management of student participation and teaching feedback, may address these limitations.

**Objective:**

This study aims to develop and evaluate a BOPPPS-based micro-lecture teaching system for orthopaedic postgraduates, to improve teaching quality, enhance students’ clinical competencies, and provide evidence-based insights for teaching reform.

**Methods:**

Forty-eight first-year surgical postgraduates in a residency program were randomly divided into experimental and control groups (n = 24 per group). The experimental group received micro-lecture instruction integrated within the BOPPPS framework, while the control group received ordinary teaching. Both groups were assessed using the Mini-Clinical Evaluation Exercise and Direct Observation of Procedural Skills after clinical training. A questionnaire survey was conducted to gauge students’ satisfaction with the BOPPPS-based micro-lectures.

**Results:**

The experimental group showed significantly better clinical diagnostic and treatment abilities than the control group across most domains (*p* < 0.007). No significant differences were identified in humanistic care (*p* = 0.015), providing detailed information and informed consent (*p* = 0.190), or communication skills with patients (*p* = 0.209) after Bonferroni correction. Performance in preoperative preparation reached the threshold of significance (*p* = 0.005). The experimental group outperformed the control group in eight other clinical practice indicators (*p* < 0.005).

## Introduction

Mastering the knowledge and skills of orthopaedics is challenging for postgraduates due to the discipline’s broad scope and complexity. In China, clinical education for orthopaedic postgraduates remains primarily teacher-centred and face-to-face, causing low efficacy in conveying knowledge and skills to students [1,2]. Previous teaching practices have suggested the application of digital micro-lectures as a supplementary approach for face-to-face lectures. Delivered as short videos demonstrating one simple, self-contained knowledge or skill, digital micro-lectures offer advantages in flexibility, visualisation, and portability. When supported by multimedia and internet platforms, micro-lectures have been shown to promote students’ initiative and efficiency of independent learning [3]. However, the educational potential of micro-lectures is often underutilized, for they are frequently used as standalone supplements rather than being systematically connected with the teacher-centred, conventional lectures.

In search of effective solutions, the bridge-in, learning objective, pre-test, participatory learning, post-assessment, and summary (BOPPPS) teaching model was proposed by the Instructional Skills Workshops (ISW) in North American universities. As a student-centred teaching framework, the BOPPPS teaching model emphasises a closed-loop management of student participation and teaching feedback by organising the teaching process into six recyclable stages: bridge-in, learning objective, pre-test, participatory learning, post-assessment and summary [4–6]. Previous studies have confirmed its efficacy in improving knowledge comprehension and students’ satisfaction in medical postgraduates [7].

By practicing the BOPPPS teaching model, multiple micro-lectures are interconnected to construct a comprehensive and mature teaching system for orthopaedic research postgraduates. This approach facilitates students’ systematic mastery of subject knowledge while strengthening their competency through teaching methods that focus more on interactive student participation [8–10]. Therefore, the BOPPPS model may provide a viable, structured framework that could enable micro-lectures to function as key components rather than supplements. However, despite the growing application of micro-lectures and the BOPPPS model in medical education, evidence remains limited regarding their systematic integration in orthopaedic postgraduate training. It remains unclear whether combining micro-lectures with the BOPPPS model can overcome the limitation of micro-lectures’ isolated application, thereby improving the students’ clinical competency outcomes.

Therefore, our study aims to construct and evaluate a BOPPPS-based micro-lecture teaching system, building upon the existing teaching experiences at our centre. Complex orthopaedic concepts and procedures were reorganised into a series of engaging, vivid, and intuitive micro-lectures, forming a logically structured teaching system. Multiple teaching and assessment strategies, including Mini-Clinical Evaluation Exercise (Mini-CEX), Direct Observation of Procedural Skills (DOPS), and Problem-Based Learning (PBL), were utilised to achieve the intended course objectives. This study seeks to provide preliminary evidence for improving teaching quality and students’ clinical competency in orthopaedic graduate education, and to inform future clinical teaching reform.

## Methods

### Research subjects

This study was designed as an exploratory, single-centre, randomised educational study. A total of 48 first-year surgical postgraduate students who rotated in the Department of Orthopaedics within the resident physician training base of China-Japan Friendship Hospital between July 2022 and June 2023 were enroled. Due to the fixed number of first-year postgraduate residents enroled during the study period and the exploratory nature of the study, all eligible students were included, and no power calculation was performed. All students who participated met the admission criteria, especially the score requirement on the National Postgraduate Entrance Examination set by the hospital’s postgraduate programme. These students were randomly divided into experimental and control groups, with 24 students in each group, using a random number table. Given the educational nature of the intervention, allocation concealment and blinding were not feasible. Standardised evaluation tools were used to reduce potential assessment bias. General information such as the student ID, age, gender, nationality, and professional background, was recorded. The research protocols were approved by the Ethics Committee of the China-Japan Friendship Hospital [approval number 2024-KY-357]. The research protocols follow all relevant guidelines and comply with the standards of the Helsinki Declaration. Written informed consent was obtained from all participants before enrolment. For the anonymous questionnaire survey, completion and submission of the questionnaire were considered to imply consent, as approved by the ethics committee.

### Research content

Students in the experimental group (ESs) adopted the micro-lecture teaching system based on the BOPPPS teaching model.

Bridge-in: ESs were required to complete a series of micro-lectures on relevant basic orthopaedic knowledge through an online-chatting group at least two weeks in advance, to establish a basic understanding of orthopaedics.

Learning objectives: ESs were also required to understand the learning objectives that should be acquired during their rotation. Meanwhile, preceptors were required to clarify the interconnection between key teaching points by completing a teaching design form based on the BOPPPS teaching model, to achieve teaching uniformity among preceptors.

Pre-test: ESs were allowed to begin the orthopaedic rotation only after passing a pre-clinical learning test on the previous micro-lectures. The test was also delivered through the same online chatting group.

Participatory learning: During rotation, preceptors were asked to deliver a new set of micro-lectures based on the design form mentioned previously. These new micro-lectures were delivered four times (eight hours in total), with content including common diseases, physical examinations, and operating skills. Self-studying was also required for ESs during their spare time. Other diverse teaching methods were adopted to facilitate participatory learning, including Mini-CEX, DOPS, Case-Based Learning (CBL), PBL, and Team-Based Learning (TBL); Preceptors mainly demonstrated typical cases and interspersed teaching with content related to humanities and doctor-patient communication.

**Figure 1.**
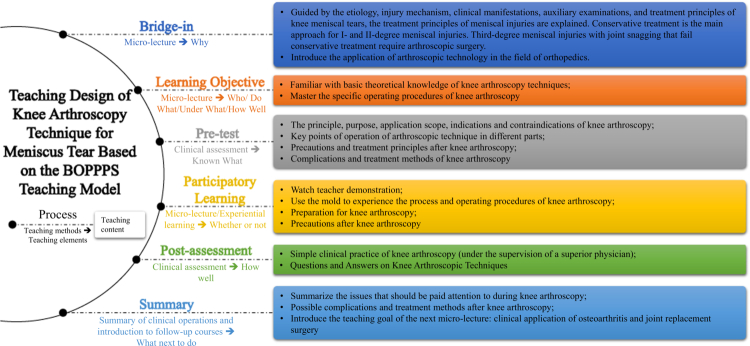
Teaching design of knee arthroscopy technique for meniscus tear based on the BOPPPS teaching model.

Post-assessment and summary: Preceptors were required to provide feedback on students' strengths and weaknesses after Mini-CEX and DOPS, point out any missed issues, correct errors, and provide individualised guidance and assistance based on each student's performance. This was to promote ESs' understanding and application of knowledge, help them develop clinical thinking, improve practical skills, and reinforce doctor-patient communication. ESs were also reminded to start learning for subsequent courses during this process. Figure 1 demonstrates the clinical teaching design of the improved BOPPPS micro-lecture model for teaching knee arthroscopy techniques for meniscus tear.

Ordinary teaching methods were employed for students in the control group (CSs). CSs entered orthopaedics without bridge-in lectures or exams. They underwent a four-session course (eight hours in total) of PBL combined with multimedia teaching. Teaching strategies, such as Mini-CEX, DOPS, CBL, PBL, and TBL, were also employed in the control group, reflecting standard postgraduate teaching practice. During these sessions, common orthopaedic diseases were exemplified, and instruction was provided on musculoskeletal examination and skill training.

Both ESs and CSs completed a two-month clinical learning period in the orthopaedic department.

### Evaluation criteria

Teaching effectiveness was evaluated through assessments of clinical diagnostic ability, clinical decision-making capacity, and clinical skills. In addition, an anonymous questionnaire survey was conducted with the ESs to understand their satisfaction with the BOPPPS-based micro-lecture teaching system. The questionnaire was developed through the Delphi method by a panel of orthopaedic surgeons and educators from the hospital’s education department. Content validity was assessed by two senior orthopaedic experts. Internal consistency reliability was assessed using the Kuder-Richardson Formula (KR-20).

Clinical diagnostic ability and capability of clinical decision-making were assessed in both groups after two months of rotation using simulated patient consultation. A common orthopaedic disease was selected randomly from the question bank of our department. Two experienced clinicians reviewed the selected diseases to verify equivalent complexity for all students. A simulated patient consultation was conducted afterwards. The content of which encompassed patient’s symptoms, medical history, and physical signs. Students from both groups were required to collect medical histories, extract diagnostic bases, and provide treatment plans. During the assessment, two preceptors from the orthopaedics department used a unified Mini-CEX scoring scale (Table 1) for evaluation. Each item was scored on a 9-point scale, with scores ranging from 1 to 3 indicating substandard performance, 4 to 6 indicating standard performance, and 7 to 9 indicating excellent performance. The average score from the two preceptors was used as the clinical competency score for each student.

**Table 1.** Mini-CEX scoring scale and DOPS scoring scale.

Clinical diagnostic and therapeutic capabilities	Scale	Clinical skills	Scale
Medical history enquiry	1~9	Familiarity with anatomy and indications	1~8
Physical examination	1~9	The ability to provide detailed information to patients and obtain consent	1~8
Humanistic care	1~9	Preoperative preparation	1~8
Clinical judgement	1~9	Appropriate analgesia or sedation anaesthesia	1~8
Communication skills	1~9	Clinical operation skills	1~8
Organisational skills	1~9	Seeking help when needed	1~8
Overall performance	1~9	Post-operative management	1~8
		Aseptic technique	1~8
		Communication skills with patients	1~8
		Consideration of patient feelings	1~8
		Overall performance of clinical operations	1~8

The practice of joint puncture was chosen for the evaluation of students’ clinical skills. Students from both groups were asked to perform a complete joint puncture procedure on a manikin. Two preceptors from the orthopaedics department used a unified DOPS scoring scale (Table 1) to evaluate students. The assessment was conducted on an 8-point scale, with scores ranging from 1 to 2 indicating below-expected performance, 3 to 4 indicating near-expected performance, 5 to 6 indicating expected performance, and 7 to 8 indicating above-expected performance. The average score from the two preceptors was used as the clinical skills score for each student.

All preceptors involved in the final evaluation did not participate in the teaching process and underwent strict training to avoid potential bias.

### Statistical analysis

All statistical indicators were expressed as mean ± standard deviation. Statistical analysis was performed using SPSS 25.0 software. For homogenous metric data, one-way analysis of variance (ANOVA) was used, while Welch's ANOVA was used for heterogeneous metric data. The chi-square test was used for count data. To control for multiple comparisons, the significance threshold was adjusted using the Bonferroni method. The significance level of *p* < 0.05 was considered statistically significant.

## Results

There were no statistically significant differences in gender (χ2 = 1.371; *p* = 0.242) or age (t = −0.351; *p* = 0.727) between the two groups of students.

### Results of Mini-CEX assessment of clinical diagnostic ability and capability of clinical decision-making between groups

The clinical diagnostic ability and capability of clinical decision-making assessment results for both groups of students are presented in Table 2. After Bonferroni correction (*α* = 0.05/7), *p* < 0.007 was considered statistically significant. The results indicate that the ESs outperformed CSs in almost all aspects, excluding humanistic care. (*p* < 0.007).

**Table 2.** Results of clinical diagnosis and treatment ability assessment of two groups of students.

Evaluation project	Group (mean ± standard deviation)		Welch *F*	*p*
	Experimental group (*n* = 24)	Control group (*n* = 24)		
Medical history enquiry	7.21 ± 1.10	5.88 ± 1.08	17.979	0.000**
Physical examination	6.88 ± 0.99	5.71 ± 1.16	14.022	0.001*
Humanistic care	6.83 ± 1.17	5.96 ± 1.23	6.375	0.015
Clinical judgement	7.33 ± 1.20	5.54 ± 0.93	33.250	0.000**
Communication skills	7.17 ± 0.82	6.13 ± 0.99	15.779	0.000**
Organisational skills	7.13 ± 0.80	6.04 ± 0.86	20.512	0.000**
Overall performance	7.38 ± 0.58	6.25 ± 1.15	18.325	0.000**

* *p* < 0.007 (Bonferroni corrected).

** *p* < 0.001 (Bonferroni corrected).

### Results of DOPS assessment of clinical skills between groups

The results of the clinical skills assessment for both groups of students are presented in Table 3. After Bonferroni correction (*α* = 0.05/11), *p* < 0.005 was considered statistically significant. The results indicate that ESs outperformed CSs in familiarity with anatomy and indications, appropriate analgesia or sedation anaesthesia, clinical operation skills, seeking help when needed, post-operative management, aseptic techniques, consideration of patient feelings, and overall performance of clinical operations (all eight items) (*p* < 0.005). The performance difference in preoperative preparation is at the threshold of significance (*p* = 0.005) after Bonferroni correction. However, there is no significant difference in the two evaluation indicators regarding the ability to provide detailed information to patients and obtain consent and communication skills with patients.

**Table 3.** Clinical skills assessment results of two groups of students.

Evaluation project	Group (mean ± standard deviation)		*F*	*p*
	Experimental group (*n* = 24)	Control group (*n* = 24)		
Familiarity with anatomy and indications	6.46 ± 0.78	5.50 ± 0.88	15.863	0.000**
The ability to provide detailed information to patients and obtain consent	6.13 ± 0.80	5.79 ± 0.93	1.773	0.190
Preoperative preparation	6.29 ± 0.91	5.54 ± 0.83	8.893	0.005
Appropriate analgesia or sedation anaesthesia	6.42 ± 0.83	5.46 ± 0.93	14.164	0.000**
Clinical operation skills	6.63 ± 0.92	5.29 ± 0.75	30.118	0.000**
Seeking help when needed	6.46 ± 1.14	5.46 ± 0.93	11.058	0.002*
Post-operative management	6.54 ± 0.78	5.67 ± 0.96	11.975	0.001*
Aseptic technique	6.42 ± 0.78	5.50 ± 0.78	16.665	0.000**
Communication skills with patients	6.00 ± 0.72	5.71 ± 0.86	1.622	0.209
Consideration of patient feelings	6.25 ± 0.79	5.50 ± 1.06	7.667	0.008*
Overall performance of clinical operations	6.54 ± 0.83	5.58 ± 0.72	18.241	0.000**

* *p* < 0.005 (Bonferroni corrected).

** *p* < 0.001 (Bonferroni corrected).

### Satisfaction survey results of experimental group students

At the end of the rotation, 24 questionnaires were distributed to ECs, and 24 valid questionnaires were collected, resulting in a 100% response rate. The questionnaire demonstrated moderate internal consistency, with a KR-20 coefficient of 0.667. The survey results are presented in Table 4, showing that 23 ECs preferred this new teaching method to the ordinary teaching method.

**Table 4.** Evaluation of the implementation effectiveness of the new teaching method by 24 students in the experimental group [*n* (%)].

Students’ evaluations of the implementation of new teaching methods	YES	NO
Whether it is more conducive to improving the ability to collect medical history.	22 (91.7)	2 (8.3)
Whether it is more conducive to cultivating clinical reasoning skills.	21 (87.5)	3 (12.5)
Whether it is more conducive to enhancing clinical procedural skills.	20 (83.3)	4 (16.7)
Whether it is more beneficial for increasing awareness of humanistic care.	24 (100)	0 (0)
Whether it is more advantageous for enhancing doctor-patient communication skills.	23 (95.8)	1 (4.2)
Whether it is more conducive to recognising one's own shortcomings accurately.	24 (100)	0 (0)
Whether it is more conducive to fostering self-directed learning abilities.	22 (91.7)	2 (8.3)
Whether it is more effective in stimulating interest in learning.	22 (91.7)	2 (8.3)
Whether it increases psychological pressure in learning.	4 (16.7)	20 (83.3)
Whether it is more popular.	23 (95.8)	1 (4.2)

## Discussion

The BOPPPS model was first proposed in 1978 and has been widely praised in Canada and many other countries in North America for its simplicity and ease of use in teaching. The core concept of the BOPPPS teaching model emphasises comprehensive student engagement and providing timely feedback on learning outcomes, thereby enhancing preceptors' instructional design and organisation, reinforcing the student-centred nature of classroom teaching, and promoting participation and interaction during teaching [11]. In recent years, the BOPPPS teaching model has achieved promising teaching results in domains such as vocational skills training, theoretical teaching, experimental teaching, and technical operation training. Research conducted in Chinese universities has shown significant effects of the BOPPPS teaching model on cultivating both the students' clinical abilities and preceptors' teaching skills, strengthening the evaluation system, while steadily improving the quality of practical teaching [4,12]. Furthermore, the integration of information systems with the BOPPPS teaching model can enhance student engagement throughout the entire learning process, thereby enhancing students’ capacity for self-directed learning [2]. Relying on the digitalised nature of the BOPPPS teaching model, a comprehensive lecture system for orthopaedic postgraduates can be constructed by connecting multiple micro-lectures, thus helping students master disciplinary knowledge systematically.

In clinical teaching for orthopaedic postgraduates, the acquisition of procedural competency requires high level of spacial reasoning. Key subjects include medical imaging, musculoskeletal anatomy, physical examination and the performance of specialised procedural techniques. The acquisition of these skills highly depends on two key factors: preceptors’ direct demonstration and students’ own observations and sustained practices [4,13]. Verbal instruction alone is considered insufficient. To address these challenges, online digitalised micro-lectures can be employed. The vivid presentation of condensed knowledge in micro-lectures enhances students’ understanding and promotes independent learning, thereby optimising the clinical teaching model and process for orthopaedic postgraduates [1,8].

On the other hand, while the BOPPPS-based micro-lecture system shows potential in promoting students’ proactive learning and enhancing their ability for comprehensive analysis and clinical reasoning, it still faces several challenges and limitations [14]. To start with, standalone micro-lectures often focus on a single topic due to their ‘micro’ nature, making them primarily suitable for knowledge points self-contained and fundamental. Conversely, it proves less effective in conveying macroscopic topics with complex logical structures and substantial content volume. Poorly produced micro-lectures with isolated knowledge points and insufficient inner connections will inevitably affect teaching effectiveness. Secondly, the production of serial micro-lectures requires continuous investment and well-established protocols. The high cost of production limits micro-lectures to a supplementary role rather than a viable alternative to traditional teacher-centred lectures.

Lastly, our result indicates that the BOPPPS-based micro-lecture system needs to be supplemented by hands-on experiences and practices, especially in skill-oriented subjects. Our results show no significant differences between ESs and CSs in doctor-patient communication skills (the skill of general communication, the obtainment of informed consent, and Humanistic care), especially after Bonferroni correction. This may be partially attributable to the reduced statistical power resulting from the conservative nature of the Bonferroni correction, particularly given the small sample size. Moreover, unlike other practical skills, such as joint puncture or physical examination, communication skills require complex logical thinking and real-world experiences that cannot be demonstrated in a short time. Therefore, the acquisition of communication skills should be considered unsuitable for the micro-lecture system. However, Yang et al. addressed this limitation by combining the BOPPPS teaching model with the situation‐background‐assessment‐recommendation (SBAR) communication mode [15]. SBAR, proposed by the World Health Organisation, is a standardised step-by-step communication strategy. By replacing micro-lectures with in-class SBAR practice, students in the experiment group showed significant improvement compared to the control group. This outcome not only confirms the necessity of in-class teaching sessions, such as in-class discussions, role-playing, and practicing on mannequins, but also shows the potential of applying the BOPPPS model for teaching macroscopic subjects, thereby demonstrating its fundamental blended nature. Therefore, it is recommended that future studies prioritise optimising micro-lecture production protocol to enhance both quality and quantity. In addition, personalised teaching approach based on real-world competency is yet to be explored for integrated teaching quality.

Clinical education for orthopaedic postgraduates faces similar challenges across China and other countries, including increased knowledge complexity, lack of standardised and validated curricula, and restricted training periods [16]. In response, various student-centred, technology-enhanced teaching strategies, including video-based microlearning and the BOPPPS model, have emerged [17,18]. Previous studies have demonstrated the effectiveness of both micro-lectures and BOPPPS model in promoting students’ engagement and training outcomes separately in global contexts [11,19]. Building on this evidence, our study combined micro-lectures within the BOPPPS framework in the context of Chinese orthopaedic postgraduate training. The observed improvement in students’ clinical competency may not be context-specific but rather holds potential applicability across diverse medical education settings. Nevertheless, further international collaboration should be encouraged to validate these findings and promote innovation in orthopaedic postgraduate education.

The result of our study is exploratory, yet there are still several limitations. Firstly, this is a single-centre, exploratory study with a limited sample size and no power calculation was performed, which may cause potential sample and selection bias. This is primarily because the number of students allocated to our training base each year by universities is limited. The exclusion of senior residents, combined with the need to control possible bias from extended research duration, contributes to the difficulty of building large cohort. Due to the educational nature of the intervention, the lack of allocation concealment and blinding may have introduced performance and assessment bias. Standardised assessment tools, such as the Mini-SEX and DOPS, were used to minimise potential bias. As such, the external validity of these results to other settings may be reduced. Future studies may adopt a multi-centre design for a sufficient sample size. Secondly, although well-trained standardised patients and manikins were used to achieve assessment consistency between groups, the validity of assessments may still be influenced by the difference between the diseases chosen for each student, which may cause potential assessment bias. Nevertheless, future studies may employ more precise methodologies to quantify test items for better uniformity. Thirdly, potential confounding variability related to student participants may cause certain bias. Although students who participated in our study met the admission criteria set by the hospital’s postgraduate programme, factors such as preceptor variability and self-motivation may differ. As this was an educational intervention, communication among students across groups could not be fully restricted, potentially leading to information sharing (e.g., learning objectives) and intergroup contamination. Future research may investigate possible solutions for the assessment and quantification of these criteria. Fourthly, because the control group was not passive comparator, the observed differences cannot be attributed solely to the application of the BOPPPS-based micro-lecture system. However, this design allows the evaluation of the educational value of BOPPPS-based micro-lecture system within a real-world teaching context.

Meanwhile, the uniformly positive responses and 100% response rate shown from the questionnaire survey may reflect certain social desirability bias inherent to self-reported measures in educational settings. Students’ awareness of participating in an educational study may have influenced their responses, even in the context of an anonymous survey. In addition, the internal consistency of the questionnaire was moderate (KR-20 = 0.667), which may be partly caused by the small sample size and the dichotomous nature of the items. Therefore, the students’ preference towards the teaching system should be interpreted with caution.

Lastly, the long-term effects of this innovative teaching approach on students have not been fully researched. This is mainly due to the strict two-month rotation plan stipulated by the Chinese standardised residency training programme. In recent years, more hospitals and universities have adopted a newly designed residency training programme specifically for orthopaedic postgraduates. The new programme features a continuous, extended training period within the orthopaedic department, offering a better chance of long-duration studies for Chinese researchers on the topic.

## Conclusion

Our study proposed a micro-lecture system based on the BOPPPS teaching model for orthopaedic postgraduate students in China. The results provide preliminary evidence that a BOPPPS-based micro-lecture system may enhance clinical diagnostic and treatment competencies among orthopaedic postgraduates. By continuously refining and innovating combined teaching methods, this approach can provide more effective teaching tools and strategies for the reform and refinement of orthopaedic postgraduate clinical teaching. Further multicenter, longitudinal studies with larger sample sizes are needed to generalise this approach across different disciplines and cultural backgrounds.

## Supplementary Material

Supplementary material Assessment Form and Questionnaire.

## Data Availability

The datasets used and/or analysed during the current study are available from the corresponding author on reasonable request.

## References

[1] LaPorte DM, Tornetta P, Marsh JL. Challenges to orthopaedic resident education. J Am Acad Orthop Surg. 2019;27(12):419–425. doi: 10.5435/JAAOS-D-18-00084 30480589

[2] Ma X, Zeng D, Wang J, et al. Effectiveness of bridge-in, objective, pre-assessment, participatory learning, post-assessment, and summary teaching strategy in Chinese medical education: a systematic review and meta-analysis. Front Med. 2022;9:975229. doi: 10.3389/fmed.2022.975229 PMC9521335 36186766

[3] Walsh S, De Villiers MR, Golakai VK. Introducing an e-learning solution for medical education in Liberia. Annals of Global Health. 2018;84(1):190–197. doi: 10.29024/aogh.21 30873817 PMC6748226

[4] Klima S, Hepp P, Löffler S, et al. A novel phased-concept course for the delivery of anatomy and orthopedics training in medical education. Anatomical Sciences Education. 2017;10(4):372–382. doi: 10.1002/ase.1675 28002644

[5] Muttappallymyalil J, Mendis S, John LJ, et al. Evolution of Technology in Teaching: Blackboard and beyond in Medical Education. Nepal J Epidemiol. 2016;6(3):588–592. doi: 10.3126/nje.v6i3.15870 27822404 PMC5082488

[6] Prekeges J. Workshop on instructional skills in the clinical setting. J Nucl Med Technol. 2018;46(2):96–98. doi: 10.2967/jnmt.118.209213 29599402

[7] Li S, Wei W, Li X, et al. Impacts of blended learning with BOPPPS model on Chinese medical undergraduate students: a comprehensive systematic review and meta-analysis of 44 studies. BMC Med Educ. 2024;24(1):914. doi: 10.1186/s12909-024-05917-x 39180016 PMC11344447

[8] Dixon KA, Cotton A, Moroney R, et al. The experience of sessional teachers in nursing: a qualitative study. Nurse Educ Today. 2015;35(11):1097–1101. doi: 10.1016/j.nedt.2015.06.008 26169288

[9] Hu K, Ma RJ, Ma C, et al. Comparison of the BOPPPS model and traditional instructional approaches in thoracic surgery education. BMC Med Educ. 2022;22(1):447. doi: 10.1186/s12909-022-03526-0 35681190 PMC9185859

[10] Liu XY, Lu C, Zhu H, et al. Assessment of the effectiveness of BOPPPS-based hybrid teaching model in physiology education. BMC Med Educ. 2022;22(1):217. doi: 10.1186/s12909-022-03269-y 35354465 PMC8966603

[11] Cooke PC, Hajamohideen N, Gooneratne H. Developing a blended learning postgraduate teaching programme in anaesthesia: pandemic and beyond. Postgrad Med J. 2022;98(1161):559–563. doi: 10.1136/postgradmedj-2021-140155 37066504

[12] Yang Y, You J, Wu J, et al. The effect of microteaching combined with the BOPPPS model on dental materials education for predoctoral dental students. AADS Proceedings. 2019;83(5):567–574. doi: 10.21815/JDE.019.068 30858273

[13] Kogan M, Klein SE, Hannon CP, et al. Orthopaedic education during the COVID-19 pandemic. J Am Acad Orthop Surg. 2020;28(11):e456–e464. doi: 10.5435/JAAOS-D-20-00292 32282439 PMC7195844

[14] Wen H, Xu W, Chen F, et al. Application of the BOPPPS-CBL Model in electrocardiogram teaching for nursing students: a randomized comparison. BMC Med Educ. 2023;23(1):987. doi: 10.1186/s12909-023-04983-x 38129836 PMC10740289

[15] Yang D, Ai X, Cai M. Research on doctor‐patient communication teaching for oncology residents: a new teaching model. Evaluation Clinical Practice. 2025;31(5):e14217. doi: 10.1111/jep.14217 PMC12239543 39431536

[16] Myers TG, Marsh JL, Nicandri G, et al. Contemporary issues in the acquisition of orthopaedic surgical skills during residency: competency-based medical education and simulation. J Bone Jt Surg. 2022;104(1):79–91. doi: 10.2106/JBJS.20.01553 34752441

[17] Román‐Sánchez D, De‐La‐Fuente‐Rodríguez JM, Paramio A, et al. Evaluating satisfaction with teaching innovation, its relationship to academic performance and the application of a video‐based microlearning. Nurs Open. 2023;10(9):6067–6077. doi: 10.1002/nop2.1828 37221960 PMC10416003

[18] Zhang SL, Ren SJ, Zhu DM, et al. Which novel teaching strategy is most recommended in medical education? a systematic review and network meta-analysis. BMC Med Educ. 2024;24(1):1342. doi: 10.1186/s12909-024-06291-4 39574112 PMC11583476

[19] De Gagne JC, Park HK, Hall K, et al. Microlearning in health professions education: scoping review. JMIR Med Educ. 2019;5(2):e13997. doi: 10.2196/13997 31339105 PMC6683654

