# Supplementary material for: A BOPPPS-based micro-lecture teaching intervention for orthopaedic postgraduates in China
Source: Med Educ Online. 2026 Jan 13;31(1):2616194. doi: 10.1080/10872981.2026.2616194 (PMC12802514; doi:10.1080/10872981.2026.2616194)
Supplement: Supplementary material — Assessment Form and Questionnaire. [file ZMEO_A_2616194_SM1103.docx]

Dear Participants,

Welcome to the clinical assessment test project. Please review the following carefully:

1. Informed consent: By participating, you confirm that you fully understand the purpose, content, and your rights regarding this test, and voluntarily agree to participate. Your participation is entirely voluntary, and you have the right to withdraw at any time without any negative consequences.

2. Data confidentiality and privacy protection: We guarantee that all collected data will solely serve the research purposes of this project and will be strictly kept confidential and protected in accordance with relevant laws and regulations. Your personal information and test results will not be disclosed to any third party.

3. Feedback and guidance: After the test, two assessors will provide detailed feedback and guidance to help you identify your strengths and areas for improvement. This feedback will serve as a valuable reference for your personal and professional development.

4. Contact information: If you have any questions or concerns about the test or project, please feel free to contact our project team anytime. We are committed to answering your questions and providing the necessary assistance.

Thank you again for your participation and support! We believe that through your contributions, we can collectively advance clinical skills education and training, thereby positively contributing to the enhancement of medical service quality. Please sign and date below to indicate your full understanding and agreement to participate in this project.

Signature: __________ Date: __________

Mini-CEX Rating Scale

（clinical diagnosis and treatment ability assessment）

**Student Name**: ___________________ **Assessment Date**: ___________________

**Assessors**: ___________________ & ___________________

| **Evaluation project** | **Rating Criteria** | **Assessor 1 Score** | **Assessor 2 Score** | **Average Score** |
| --- | --- | --- | --- | --- |
| **History Taking** | 1-3: Information collection is incomplete, with crucial details missing. |  |  |  |
|  | 4-6: Information collection is generally comprehensive, covering key aspects. |  |  |  |
|  | 7-9: Information collection is thorough, detailed, and covers all crucial points. |  |  |  |
| **Physical Examination** | 1-3: Examination steps are disorganized, with key signs not assessed. |  |  |  |
|  | 4-6: Examination steps are generally standardized, with key signs assessed. |  |  |  |
|  | 7-9: Examination steps are standardized, thorough, and all key and auxiliary signs are assessed. |  |  |  |
| **Humanistic Care** | 1-3: Lacks understanding and empathy for patient emotions. |  |  |  |
|  | 4-6: Demonstrates basic concern and understanding for the patient. |  |  |  |
|  | 7-9: Fully demonstrates empathy, effectively reassuring the patient. |  |  |  |
| **Clinical Judgment** | 1-3: Diagnostic approach is unclear, with incorrect judgments. |  |  |  |
|  | 4-6: Diagnostic approach is generally clear, with basically correct judgments. |  |  |  |
|  | 7-9: Diagnostic approach is clear, judgments are accurate, and considerations are thorough. |  |  |  |
| **Communication Skills** | 1-3: Communication is poor, making it difficult to understand patient needs. |  |  |  |
|  | 4-6: Communication is generally smooth, effectively conveying information. |  |  |  |
|  | 7-9: Communication is fluent, effectively guiding the patient and gaining trust. |  |  |  |
| **Organizational Skills** | 1-3: The diagnostic process is chaotic and lacks organization. |  |  |  |
|  | 4-6: The diagnostic process is generally orderly with reasonable steps. |  |  |  |
|  | 7-9: The diagnostic process is efficient and orderly, with good time management. |  |  |  |
| **Overall Performance** | 1-3: Overall performance is poor, requiring significant improvement. |  |  |  |
|  | 4-6: Overall performance is good, with room for improvement. |  |  |  |
|  | 7-9: Overall performance is excellent, deserving praise. |  |  |  |

**Overall Comments and Suggestions**:

Assessor 1's Comments: _________________________________________________________

Assessor 2's Comments: _________________________________________________________

Please have both assessors provide fair and objective scores for each category based on the student's actual performance during the simulated patient consultation. Additionally, provide specific feedback and guidance in the "Overall Comments and Suggestions" section to help the student identify their strengths and weaknesses, further enhancing their clinical diagnosis and treatment abilities.

Clinical Skills Assessment Scoring Scale

**Student Name**: ___________________ **Assessment Date**: ___________________

**Assessors**: ___________________ & ___________________

| **Evaluation project** | **Scoring Criteria** | **Assessor 1 Score** | **Assessor 2 Score** | **Average Score** |
| --- | --- | --- | --- | --- |
| Familiarity with anatomy and indications | 1-2: Unfamiliar with anatomy and indications |  |  |  |
|  | 3-4: Somewhat familiar with anatomy and indications |  |  |  |
|  | 5-6: Familiar with anatomy and indications |  |  |  |
|  | 7-8: Very familiar with anatomy and indications, able to explain accurately |  |  |  |
| The ability to provide detailed information to patients and obtain consent | 1-2: Failed to inform the patient or obtain consent |  |  |  |
|  | 3-4: Informed the patient and obtained consent, but the explanation was not detailed |  |  |  |
|  | 5-6: Informed the patient in detail and obtained consent |  |  |  |
|  | 7-8: Informed the patient very thoroughly, explained fully, and obtained clear consent |  |  |  |
| Preoperative preparation | 1-2: Preparation was inadequate, important steps were missed |  |  |  |
|  | 3-4: Preparation was basically sufficient, but there were small omissions |  |  |  |
|  | 5-6: Preparation was sufficient and met the requirements of the procedure |  |  |  |
|  | 7-8: Preparation was very thorough, exceeding basic requirements |  |  |  |
| Appropriate analgesia or sedation anesthesia | 1-2: Did not use or used analgesia/sedation improperly |  |  |  |
|  | 3-4: Used analgesia/sedation reasonably, but with some issues |  |  |  |
|  | 5-6: Used analgesia/sedation appropriately, meeting procedural standards |  |  |  |
|  | 7-8: Used analgesia/sedation very appropriately, enhancing patient comfort |  |  |  |
| Clinical operation skills | 1-2: Poor skills, with obvious errors |  |  |  |
|  | 3-4: Average skills, with minor errors |  |  |  |
|  | 5-6: Good skills, meeting standards |  |  |  |
|  | 7-8: Excellent skills, exceeding standards |  |  |  |
| Aseptic technique | 1-2: Used sterile technique improperly, posing a risk of infection |  |  |  |
|  | 3-4: Basically followed sterile technique, but with small omissions |  |  |  |
|  | 5-6: Strictly followed sterile technique, with standard procedures |  |  |  |
|  | 7-8: Used sterile technique very well, with no risk of infection |  |  |  |
| Seeking help when needed | 1-2: Did not seek help when needed |  |  |  |
|  | 3-4: Basically able to seek help when needed |  |  |  |
|  | 5-6: Able to seek help timely and effectively |  |  |  |
|  | 7-8: Very good at seeking and accepting help when needed |  |  |  |
| Post-operative management | 1-2: Post-procedure management was inappropriate, may have caused discomfort to the patient |  |  |  |
|  | 3-4: Post-procedure management was basically reasonable, but with small omissions |  |  |  |
|  | 5-6: Post-procedure management was reasonable and met standards |  |  |  |
|  | 7-8: Post-procedure management was very good, exceeding standards |  |  |  |
| Communication skills with patients | 1-2: Poor communication skills, difficult to communicate effectively with the patient |  |  |  |
|  | 3-4: Average communication skills, basically able to communicate with the patient |  |  |  |
|  | 5-6: Good communication skills, able to communicate effectively with the patient |  |  |  |
|  | 7-8: Excellent communication skills, greatly enhancing the patient experience |  |  |  |
| Consideration of patient feelings | 1-2: Did not consider the patient's feelings, the procedure may have caused discomfort to the patient |  |  |  |
|  | 3-4: Basically considered the patient's feelings, but with small omissions |  |  |  |
|  | 5-6: Fully considered the patient's feelings, the procedure was comfortable |  |  |  |
|  | 7-8: Very attentive to the patient's feelings, the procedure was extremely comfortable |  |  |  |
| Overall performance of clinical operations | 1-2: Poor overall performance, significant improvement needed |  |  |  |
|  | 3-4: Average overall performance, with room for improvement |  |  |  |
|  | 5-6: Good overall performance, meeting standards |  |  |  |
|  | 7-8: Excellent overall performance, exceeding standards |  |  |  |

**Overall Evaluation and Suggestions**:

Assessor 1's Comments: ___________________________________________________________

Assessor 2's Comments: ___________________________________________________________

Please have the two assessors provide fair and objective scores for each item based on the student's actual performance during the joint puncture model procedure, and provide specific feedback and guidance in the "Overall Evaluation and Suggestions" section to help the student identify

**Evaluation of the Implementation Effectiveness of the New Teaching Method**

**（Experimental Group）**

| **Students’ evaluations of the implementation of new teaching methods** | **YES** | **NO** |
| --- | --- | --- |
| Whether it is more conducive to improving the ability to collect medical history. |  |  |
| Whether it is more conducive to cultivating clinical reasoning skills. |  |  |
| Whether it is more conducive to enhancing clinical procedural skills. |  |  |
| Whether it is more beneficial for increasing awareness of humanistic care. |  |  |
| Whether it is more advantageous for enhancing doctor-patient communication skills. |  |  |
| Whether it is more conducive to recognizing one's own shortcomings accurately. |  |  |
| Whether it is more conducive to fostering self-directed learning abilities. |  |  |
| Whether it is more effective in stimulating interest in learning. |  |  |
| Whether it increases psychological pressure in learning. |  |  |
| Whether it is more popular. |  |  |
